# Rooibos (*Aspalathus linearis*) extract enhances boar sperm velocity up to 96 hours of semen storage

**DOI:** 10.1371/journal.pone.0183682

**Published:** 2017-08-24

**Authors:** José Luis Ros-Santaella, Eliana Pintus

**Affiliations:** Department of Veterinary Sciences, Faculty of Agrobiology, Food and Natural Resources, Czech University of Life Sciences Prague, Prague, Czech Republic; Zhejiang University College of Life Sciences, CHINA

## Abstract

Rooibos (*Aspalathus linearis*) is a native shrub from South African fynbos and has become very popular in the last decades for its antioxidant and medicinal attributes. Several studies have shown its beneficial properties in numerous cell lines, but to date, the *in vitro* effects of rooibos extract on sperm cells are still unknown. In this study, boar semen was supplemented with four concentrations both of fermented and unfermented rooibos extracts during 96 h of liquid storage at 17°C. The effects of rooibos extracts on sperm velocity, membrane integrity, and acrosomal status were evaluated at 2 h, 48 h, and 96 h of semen storage. Overall our results indicate that rooibos extract enhances sperm velocity, protects the acrosome structure, and tends to preserve the membrane integrity during semen storage. Although the unfermented rooibos showed higher total polyphenol content and total antioxidant capacity than the fermented one, the latter had better effects on sperm velocity leading to, for instance, an increase of 30% in the rectilinear velocity (VSL) at 48 h compared to the control group. Taking into account the different storage times, we established a suitable range of extracts concentrations to be used in boar semen. The rooibos extract ought to be considered as a powerful and natural source of antioxidants for the preservation of boar semen.

## Introduction

Rooibos (*Aspalathus linearis*) is an endemic shrub (Family: Fabaceae) from South African fynbos. In the last two decades, it has become very popular and widely exported for its antioxidant and medicinal properties [1]. Its popularity as a tea is also due to the absence of alkaloids or stimulants and the low tannin content, which makes it a healthy beverage [2]. Rooibos tea is usually distributed in two types: fermented and unfermented. Flavonoids are the major chemical compounds found in rooibos leaves and stems being the aspalathin (an exclusive flavonoid of this plant) the most abundant in the unfermented form [3]. Thus, it has been widely reported a higher total polyphenol content (TPC) and total antioxidant capacity (TAC) in the unfermented rooibos than in the fermented one [3, 4]; nevertheless, the concentration of some flavonoids compounds (e.g., flavonols) is higher in the fermented rooibos [3]. The potential health benefits of both fermented and unfermented rooibos infusions or extracts have been reported both in *in vivo* and *in vitro* experiments (reviewed in: [1, 5, 6]).

Recently, the positive effects of rooibos as a free radical scavenger have been tested in several cell lines such as osteoblasts [7], cardiomyocytes [8], and skin cells [9]. To date, its effects on reproductive function have been mainly tested on rats fed with rooibos aqueous extracts. Thus, an increase in the endometrium thickness was observed when animals were treated with the extracts [10]. In the male, Awoniyi *et al*. [11] found a higher activity of the superoxide dismutase (SOD) as well as a decrease in lipid peroxidation (LPO) in the testicular tissue of oxidative stress-induced rats. Moreover, rooibos extracts showed positive effects on sperm concentration, viability, and the percentage of motile sperm [12, 13]. On the other hand, Opuwari and Monsees [14] demonstrated that rooibos aqueous extracts possess anti-androgenic properties when were added to TM3 Leydig cells from immature mice testes. Until now, there are no studies about the *in vitro* effects of rooibos extract on sperm function.

Nowadays, more than 99% of artificial inseminations (AIs) in the pig breeding industry are performed using extended semen in the liquid state and stored at 15–20°C [15]. The low amount of cholesterol and the high proportion of unsaturated phospholipids in the boar sperm membrane promote the high susceptibility of sperm to the cold shock and thus, to the cryopreservation process (reviewed in: [16]). Furthermore, boar sperm membrane is characterized for a high content in polyunsaturated fatty acids, which serve as a preferred substrate for reactive oxygen species (ROS) generation leading to LPO [17, 18] during semen liquid-storage. In order to palliate the effects of large amounts of ROS on sperm function (e.g., sperm velocity), several studies have tested the effects of some additives in boar semen extenders obtaining different results (reviewed in: [19]). In general, these additives considerably increase the final price of AI doses whereas the use of natural products (e.g., plant extracts) can also improve sperm function and fertilization ability [20]. Thus, due to their high tendency to produce free radicals, the boar sperm are a good model to test the effects of rooibos extract on male gametes.

The main goal of the present study was to evaluate the effects of both fermented and unfermented rooibos extracts on boar sperm velocity, membrane integrity, and acrosomal status up to 96 h of liquid semen storage. Because rooibos acts as a free radical scavenger (see references above), we expect to find positive effects on several parameters related to sperm function during boar semen storage. To test our hypothesis, we used semen samples from fifteen boars and four extracts concentrations both of fermented and unfermented rooibos. Moreover, we determined the TPC and TAC of each extract. As a natural and cheap source of antioxidants, the rooibos aqueous extract might be a good alternative to the common additives used on boar semen.

## Materials and methods

All of the reagents were purchased from Sigma-Aldrich (Prague, Czech Republic), unless otherwise indicated.

### Preparation of rooibos extracts

The extracts were prepared from fermented and unfermented rooibos (Oxalis, spol. s.r.o., Slušovice, Czech Republic) by adding 100 ml of boiling deionized water to 0.2 g of loose rooibos (leaves and stems) and steeped for 10 min (stock solution: 0.2% mass/volume, m/v). Afterwards, the extracts were filtered with a paper filter (Whatman n° 4). Four aliquots (1 ml) of each extract were frozen (−80°C) for posterior analyses to determine the TPC and TAC. The final extracts showed a pH around 6 at room temperature and were adjusted to pH 7 (NaOH 1M), which it is within the optimal range for boar semen. Then, the extracts were used to prepare the semen extender (Solusem^®^) following the manufacturer’s instructions (AIM Worldwide, Vught, Netherlands). After that, four different concentrations, both for the fermented and unfermented rooibos (FR and UR, respectively), were made by serial dilutions from the stock solution with the semen extender. The serial dilutions were as follows: 1:1 (0.1% m/v; R1), 1:6 (0.033% m/v; R2), 1:18 (0.011% m/v; R3), and 1:54 (0.004% m/v; R4). This range of concentrations was chosen based on our previous trials.

### Determination of TPC and TAC of the rooibos extracts

The TPC and TAC were determined spectrophotometrically (Hach DR 3900, Düsseldorf, Germany) as previously described [21, 22]. Briefly, for the TPC assay 500 μl of Folin-Ciocalteau reagent (0.2 N) and 400 μl of Na_2_CO_3_ solution (7.5% m/v) were added to 100 μl of deionized water (blank), gallic acid standards (2–20 μg/ml) or rooibos samples. Absorbance was measured at 765 nm after 2 h of sample incubation at room temperature in the dark. Results were expressed as gallic acid equivalents per ml of aqueous extract (μg/ml) and per g of dry leaf (mg/g). The TAC assay is based on the decolourization of the 2,2’-azino-bis-(3-ethylbenzothiazoline-6-sulfonic acid) radical cation (ABTS˙^+^) by antioxidants according to their concentration and antioxidant capacities. Briefly, 20 μl of rooibos extract was added to 800 μl of reagent 1 (acetate buffer 0.4 M, pH 5.8) and 80 μl of reagent 2 (ABTS˙^+^ in acetate buffer 30 mM, pH 3.6). Five minutes after mixing, absorbance was measured at 660 nm. Standard curve was established using known concentrations (0.25–2 mM) of 6-hydroxy-2,5,7,8-tetramethylchroman-2-carboxylic acid (Trolox). The TAC was expressed in μmol/l (Trolox equivalents).

### Semen collection and processing

Commercial sperm doses from 15 boars of different genetic lines (i.e., Czech Landrace, Pietrain, and Prestice Black-Pied) were purchased from an animal breeding company (Chovservis, a.s., Hradec Králové, Czech Republic). The ejaculates were collected by the gloved hand method, filtered through gauze to remove gel particles, and diluted with Solusem^®^ extender. All semen samples showed a minimum of 75% motile sperm. For each work session, we used the semen from three boars to make a pool in order to reduce the effects of individual male variability. Then, the sperm concentration was adjusted to 40 × 10^6^ sperm/ml with the extender. Afterwards, the semen pool was split into nine test tubes. One of the tubes was left as control and diluted with the extender (v/v), while the other tubes were diluted with the extender supplemented with the different rooibos extracts (v/v) mentioned above. Thus, the final concentrations of the rooibos extracts in the semen samples were as follows: 0.05% m/v (R1), 0.017% m/v (R2), 0.006% m/v (R3), and 0.002% m/v (R4). All the aliquots had a final sperm concentration of 20 × 10^6^ sperm/ml. All the tubes were analyzed for motility, membrane integrity, and acrosomal status after 2 h, 48 h, and 96 h of semen storage at 17°C. As a reference value, the control tube was also analyzed immediately after the dilution with the extender (0 h). The experiment was replicated five times using five different semen pools.

### Assessment of sperm motility

A sperm sub-sample (0.5 ml) was incubated at 38°C in a water bath for 15 min. After that, an aliquot of 5 μl was loaded into a Makler counter chamber (Sefi-Medical instruments, Haifa, Israel; chamber depth: 10 μm) at 38°C. Sperm motility was evaluated subjectively by estimating the percentage of motile spermatozoa to the nearest 5% and the quality of movement (QM) using a scale from 0 (lowest: no motility) to 5 (highest: progressive and vigorous movements). The sperm motility index (SMI) was calculated according to the formula: [% individual motility + (QM × 20)] / 2 [23]. Sperm kinetics was assessed by a Computer Assisted Sperm Analysis (CASA) (NIS-Elements, Nikon, Tokyo, Japan and Laboratory Imaging, Prague, Czech Republic), which consists of an Eclipse E600 tri-ocular phase contrast microscope (Nikon, Tokyo, Japan), equipped with a warming stage set at 38°C (Tokai Hit, Shizuoka, Japan), and a DMK 23UM021 digital camera (The Imaging Source, Bremen, Germany). The analysis was carried out using a 10× negative phase-contrast objective (Nikon, Tokyo, Japan). A total of eight descriptors of sperm kinetics were recorded analyzing a minimum of 400 sperm per sample: progressive motility (PM, %), average path velocity (VAP, μm/s), curvilinear velocity (VCL, μm/s), straight-line velocity (VSL, μm/s), amplitude of lateral head displacement (ALH, μm), beat-cross frequency (BCF, Hz), linearity (LIN, %), and straightness (STR, %). The standard parameter settings were as follows: no. of frames acquired: 60; minimum of frames acquired: 21; frames rate: 60 Hz; no. of fields acquired: 6. Spermatozoa with a VAP < 10 μm/s were considered as immotile and those with a VAP ≥ 20 μm/s as progressive motile [24]. Sperm kinetics parameters were assessed when at least 5% of motile cells (VAP ≥ 10 μm/s) were available.

### Assessment of sperm membrane integrity and acrosomal status

For these analyses, sperm samples were directly collected from semen stored at 17°C. For the evaluation of sperm membrane integrity, samples were stained with eosin/nigrosin (Minitube, Tiefenbach, Germany). Briefly, eosin/nigrosin stain were mixed (v/v) with the semen samples for 30 s in a pre-warmed slide (38°C) before making the smears. The samples were evaluated under bright-field microscopy. Furthermore, we assessed the acrosomal status by the NAR (normal apical ridge) test under phase contrast microscopy, after diluting a sperm aliquot in glutaraldehyde solution (2%) [25, 26]. The same researcher performed each analysis. Two–hundred spermatozoa per sample were evaluated per each analysis at 400× magnification.

### Statistical analyses

All statistical analyses were performed using the SPSS 20.0 statistical software package (IBM Inc, Chicago, IL, USA). The Shapiro–Wilk and Levene’s tests were used to check data normality and homogeneity of variance, respectively. To check for differences between fermented and unfermented rooibos in the TPC and TAC, Student’s *t*–test or Mann–Whitney *U* test were used. The repeated measures ANOVA or Friedman tests were used to check for differences in sperm parameters in the control group during the different storage times. We used a generalized linear model (GZLM) to analyze the effects of the treatments and storage times on sperm variables. Data are expressed as mean ± standard deviation. Statistical significance was set at *P* < 0.05.

## Results

### TPC and TAC in the rooibos extracts

We found higher TPC in the UR than in the FR (54.71±3.05 mg/g vs. 43.14±4.02 mg/g, respectively; expressed as gallic acid equivalent/dry leaf mass; *P* = 0.001). In the aqueous extracts, the TPC was higher in the UR than in the FR for the stock solution and the two first extracts (R1 and R2; *P* < 0.05). On the contrary, we did not find significant differences between UR and FR in the least concentrated extracts (R3 and R4; *P* > 0.05). The TPC for each extract (expressed as μg/ml) is shown in Table 1. The TAC of rooibos extracts was significantly higher (*P* < 0.05) in the UR than in the FR for the stock solution and the first extract (R1; Table 1).

**Table 1:** Total polyphenol content and total antioxidant capacity in the rooibos aqueous extracts.

Treatment	Fermented–R	Unfermented–R	*P*-value
*Total polyphenol content* ^a^			
R-stock	86.3±8	109.4±6.1	**0.009**
R1	48.9±4.6	60.7±2.6	**0.009**
R2	21.9±1.7	24.1±1.2	**0.028**
R3	12±0.7	12.4±0.4	0.172
R4	9±0.6	9.1±0.4	0.750
*Total antioxidant capacity* ^b^			
R-stock	814.7±115.3	1,008.2±107.9	**0.028**
R1	462.9±53.4	542.1±67.1	**0.047**
R2	220.8±51	232.5±48.3	0.754
R3	158±63.2	139.8±26.2	0.916
R4	140.4±45.4	137.5±41.6	0.917

^a^ = μg/ml (gallic acid equivalents);

^b^ = μmol/l (Trolox equivalents).

Bold numbers indicate significant differences (*P* < 0.05). R = rooibos. Treatments = R-stock (0.2% m/v); R1 (0.1% m/v); R2 (0.033% m/v); R3 (0.011% m/v); R4 (0.004% m/v).

### Effects of rooibos extracts on boar sperm motility

**Fig 1:**
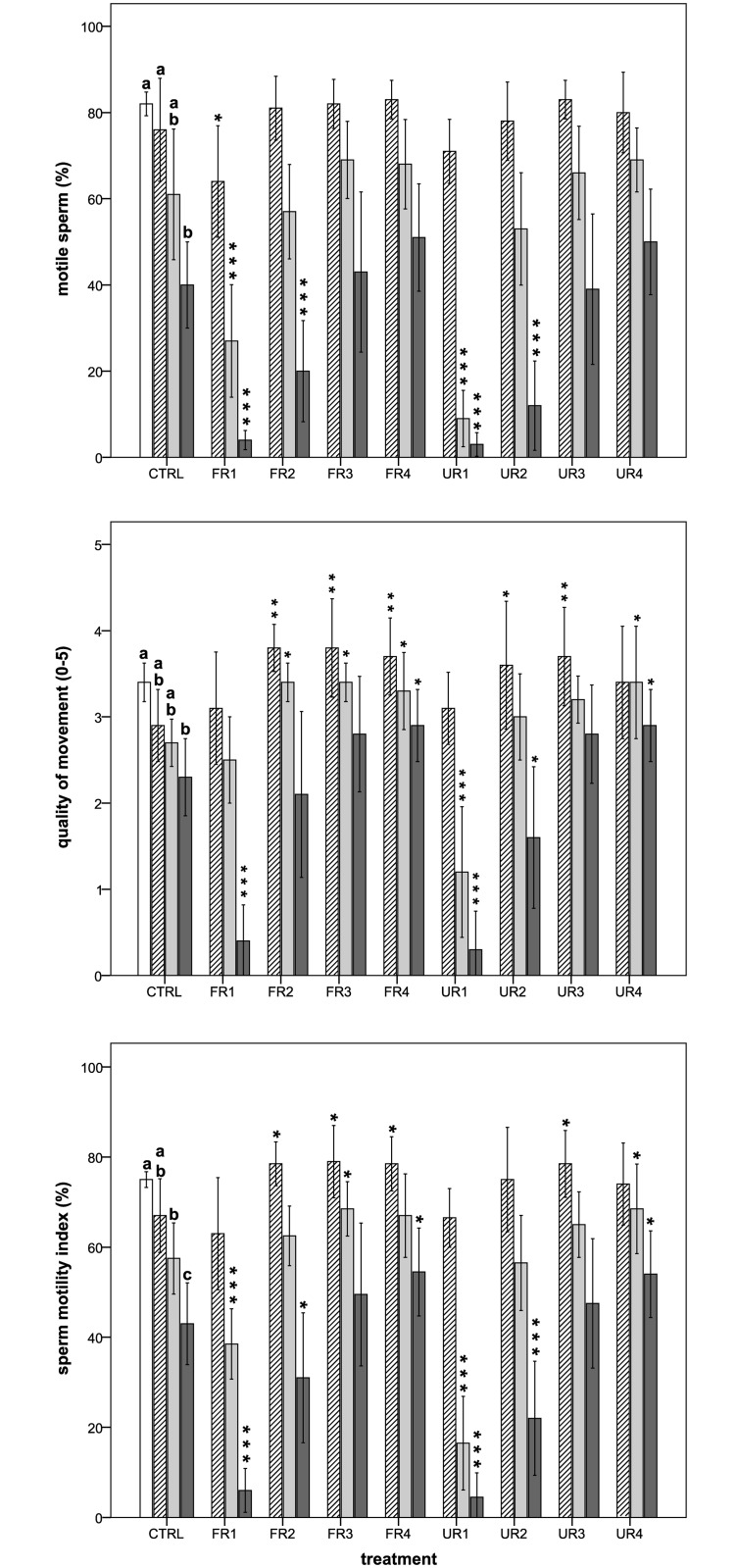
Effects of rooibos extracts on the percentage of motile sperm, quality of movement, and sperm motility index during boar semen storage. White bars = 0 h; diagonal lines bars = 2 h; light grey bars = 48 h; dark grey bars = 96 h. Different letters indicate significant differences (*P* < 0.05) among times for the control group. The asterisks indicate significant differences between the treatment and the control within each given time (*P < 0.05; **P ≤ 0.01; ***P ≤ 0.001). CTRL = control. FR = fermented rooibos. UR = unfermented rooibos. Treatments = R1 (0.05% m/v); R2 (0.017% m/v); R3 (0.006% m/v); R4 (0.002% m/v).

In general, the motility of boar sperm decreased during semen storage and was clearly affected by the different rooibos extracts (Fig 1 and Table 2). The rooibos treatments showed a significant effect (*P* < 0.05) on sperm kinetics except for ALH, BCF, and VCL parameters (*P* > 0.05). Overall, the best effects on sperm motility during semen storage were obtained with the FR treatments. For example, at 48 h of semen storage, three FR treatments (FR2, FR3, and FR4) showed positive effects on several motility parameters in contrast with the only one found in the UR (UR4).

**Table 2:** Sperm motility parameters in samples with or without rooibos extract supplementation during boar semen storage at 17°C.

Time	Treatment	PM (%)	VAP (μm/s)	VCL (μm/s)	VSL (μm/s)	ALH (μm)	BCF (Hz)	LIN (%)	STR (%)
**0 h**	Control	43.7±14.8^a^	38.5±5^a^	64.6±23.2^a^	25.9±4.3^a^	4.4±1.1^a^	9.6±2.3^a^	43.6±9.7^a^	68.7±6.4^a^
**2 h**	Control	33.1±14.4^a^	33.9±4.6^a^	58.1±13.3^a^	23.2±2.2^a^	4±1.3^a^	9.7±2.4^a^	43.1±6.4^a^^b^	71.1±9.9^a^
	FR1	27.3±15.2	31.4±6.8	48.3±12.4	26.9±5.6*	3.8±1.5	10.4±3.5	57.9±9.9*	86.3±3.3***
	FR2	41.1±13.1	36.3±5.2	55.9±14.2	28.2±2.3**	4.2±1.5	10±2.5	53.2±11	78.9±6.5**
	FR3	38.7±7.9	34.6±1.9	57.6±20.3	26.2±2.2	4.3±1.4	9.5±2.2	50.6±16.3	76.4±7.4
	FR4	42.9±6.5	36.7±4.1	62±24.8	26.6±2.7*	4.1±1	9.7±2	49.2±17.4	73.9±7.7
	UR1	30.5±11.6	31.3±4.1	47.7±9.9	25.9±3	3.7±1.3	10.1±2.7	56.7±10.6*	83.6±2.8***
	UR2	38.3±9.7	36.6±3.4	57.2±15.1	28.1±2**	4.3±1.4	10±2.4	52.1±13.3	76.7±5.4
	UR3	41.2±12.7	36.7±3.4	60.9±18.6	26.1±3.2	4.3±1.4	9.7±2.1	47.4±14.1	72.3±4.2
	UR4	33.7±10.7	33.8±4.9	59.4±24.4	24.4±3	3.9±0.9	9.6±2.1	47.3±15.3	73.5±6
**48 h**	Control	27.9±12.4^a^^b^	30.8±4.5^a^	58.2±18.6^a^	21.4±2.2^b^	3.9±1^a^	9.1±2.4^b^	41.1±8.5^a^^b^	71.1±6.6^a^
	FR1	10.1±5.6**	28±2.9	46.3±9.7	23.3±2.4	4±1.3	8.9±2.5	54.3±12.8	84.1±3.9***
	FR2	33.3±12.8	35.9±4.5*	59.3±13	28±3.7***	4.4±1.4	9.1±2.1	50.2±11.8	77.9±3.4*
	FR3	35.3±6.3	34.8±3	62.2±21.9	25±2.4*	4.1±1	9.3±2.2	44.9±12.5	72.4±3.1
	FR4	36.2±8.2	35.1±3.8	64.5±27.1	24±2.1	4.1±0.8	9.1±1.8	43.5±15.1	69.5±5.8
	UR1	N/A	N/A	N/A	N/A	N/A	N/A	N/A	N/A
	UR2	24.5±11	32±1.6	56.3±15.1	24.4±2.3	3.9±1.1	9±1.9	48.2±14.6	76.1±4.2
	UR3	32.7±8.6	33.2±4.3	61.3±25	23.2±2.1	4±0.8	9.3±2.4	43.7±12.4	70.9±3.2
	UR4	40.4±8.4*	35.2±2.6	64.9±23.9	24.1±2.1	4.2±1	9.1±2	42.8±13.9	69.9±4.5
**96 h**	Control	13.7±7.3^b^	29.3±6.3^a^	61.9±20.5^a^	21.8±4.8^a^^b^	3.5±0.6^a^	8.8±2.3^a^^b^	39±9.5^b^	73.6±3.9^a^
	FR1	N/A	N/A	N/A	N/A	N/A	N/A	N/A	N/A
	FR2	10.1±2	32.9±2.8	63.3±16.4	26.7±3.4**	3.4±0.7	9.8±2.5	46.9±9	81.1±2.3*
	FR3	25±14.7	34.8±4.5*	67.3±15.5	25.3±4.5*	3.8±1.2	9.8±1.8	40.8±10	72.4±4.8
	FR4	28.6±11.2*	34.7±5*	70.5±24.3	24±2	3.7±0.8	9.7±2	39.5±12.3	71±5.1
	UR1	N/A	N/A	N/A	N/A	N/A	N/A	N/A	N/A
	UR2	7±3.1	31.6±4.5	63.2±26.4	23.3±4.3	4±0.6	8.3±2.1	40.5±10.9	73±0.1
	UR3	22.3±10.3	30.9±1.1	63±16.7	22.6±1.8	3.5±0.9	9.4±1.5	40.2±10	73.1±2.1
	UR4	25.9±10.7*	32.2±1.7	64.7±19.3	23±1.9	3.5±0.8	9.7±1.8	40.5±11.3	72.3±3.1

^a-b^ Different superscript letters in the same column indicate significant differences (*P* < 0.05) among times for the control group.

The asterisks indicate significant differences between the treatment and the control within each given time (*P < 0.05; **P ≤ 0.01; ***P ≤ 0.001). N/A = not available. FR = fermented rooibos. UR = unfermented rooibos. Treatments = R1 (0.05% m/v); R2 (0.017% m/v); R3 (0.006% m/v); R4 (0.002% m/v). PM = progressive motility; VAP = average path velocity; VCL = curvilinear velocity; VSL = straight-line velocity; ALH = amplitude of lateral head displacement; BFC = beat-cross frequency; LIN = linearity; STR = straightness. Data are shown as mean ± standard deviation.

At 2 h of semen storage, we found positive effects on several rooibos treatments (both FR and UR) in 5 motility parameters (QM, SMI, VSL, LIN, and STR; *P* < 0.05). On the contrary, the highest concentration of FR had a negative effect on the percentage of motile sperm (*P* < 0.05), being other kinetic parameters positively increased by the same treatment (VSL, LIN, and STR; *P* < 0.05). We did not find any significant difference between the control group and the UR4 treatment in any of the motility parameters evaluated (*P* > 0.05), but, in general, the values for this treatment were higher than in the control group.

A similar trend was also observed at 48 h of semen storage, being the positive effects of rooibos extracts even more remarkable in some parameters (VSL with FR2 treatment; *P* < 0.001). Moreover, VAP was significantly higher in FR2 treatment than in the control group (*P* = 0.026). Furthermore, PM was significantly enhanced in the UR4 treatment (*P* = 0.036). On the other hand, both FR1 and UR1 treatments had detrimental effects on sperm motility. Because of that, sperm kinetics (CASA) could not have been evaluated in the UR1. We did not find any significant difference between the control group and the UR2 and UR3 treatments in any of the motility parameters evaluated (*P* > 0.05), even though most of them showed higher values than the control group.

Interestingly, the positive effects of some rooibos treatments on sperm motility persisted till 96 h of semen storage. Nevertheless, the semen samples treated with the two highest concentrations of both fermented and unfermented rooibos extracts induced a pronounced decrease in the percentage of motile sperm as well as in the SMI. For this reason, sperm kinetic parameters could not have been evaluated both for FR1 and UR1 treatments. Interestingly, both FR4 and UR4 showed around a twofold increase in PM than the control group (*P* = 0.014 and *P* = 0.049, respectively).

### Effects of rooibos extracts on sperm membrane integrity and acrosomal status

**Fig 2:**
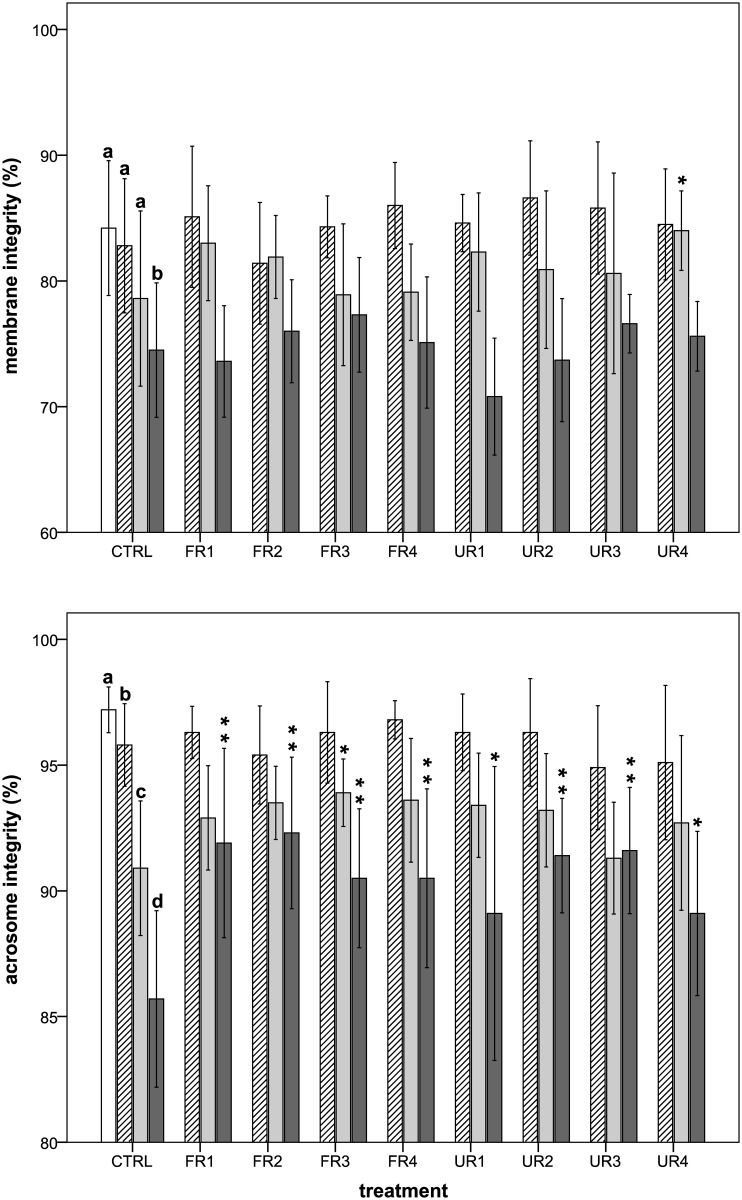
Effects of rooibos extracts on sperm membrane and acrosome integrity during boar semen storage. White bars = 0 h; diagonal lines bars = 2 h; light grey bars = 48 h; dark grey bars = 96 h. Different letters indicate significant differences (*P* < 0.05) among times for the control group. The asterisks indicate significant differences between the treatment and the control within each given time (*P < 0.05; **P ≤ 0.001). CTRL = control. FR = fermented rooibos. UR = unfermented rooibos. Treatments = R1 (0.05% m/v); R2 (0.017% m/v); R3 (0.006% m/v); R4 (0.002% m/v).

The percentage of sperm with an intact membrane decreased during semen storage (Fig 2), showing a significant decrease at 96 h (*P* < 0.05). Although there was a tendency for the sperm membrane integrity to be greater in most of rooibos treatments, we only found significant differences between the UR4 treatment and the control group at 48 h of semen storage (*P* = 0.031). In the same way, the percentage of sperm showing a normal acrosomal status was affected by storage time and significantly decreased from 2 h (*P* < 0.05; Fig 2). At 2 h of semen storage, we did not find any significant difference between the rooibos treatments and the control group (*P* > 0.05). On the other hand, the FR3 treatment showed positive and significant (*P* = 0.045) effects on acrosomal status at 48 h when compared with the control group. At 96 h of semen storage, all the rooibos treatments showed higher values than the control group (*P* < 0.05), indicating a protective effect on this organelle.

## Discussion

In the present study, for the first time to our knowledge, we found that the addition of rooibos extract to the boar semen enhances sperm velocity and preserves the acrosome integrity. These positive effects were observed from 2 h to 96 h of semen storage leading to, for example, an increase of 30% in the rectilinear velocity (VSL) at 48 h compared with the control group. Despite the fact that the UR extracts have higher TPC and TAC, the best results were obtained with the FR extracts. Based on our results and taking into account the different storage times, we established a range of extracts concentrations (4.5–11.5 μg/ml gallic acid equivalents) for boar semen.

The boar semen doses sold by breeding companies are usually diluted with commercial extenders, with the consequent reduction of seminal plasma (SP) when compared with the raw semen. Recently, it has been demonstrated the influence of boar SP in sperm quality during liquid semen storage [27], probably due to the presence of several chemical compounds with antioxidant activities [28] that may act as ROS scavengers. Thus, the addition of antioxidants to the liquid-stored boar semen has become a usual praxis in pig breeding companies, although this often increases the price of the semen doses. As an alternative to these antioxidant-additives, in the present study we tested the effects of rooibos aqueous extract added directly to the boar semen, obtaining positive effects on several sperm motility parameters, membrane integrity, and acrosomal status during sperm storage. It is important to bear in mind that around 99% of AIs are accomplished using extended semen and 85% are conducted within the first two days after semen collection (reviewed in: [19]). According to these facts, the positive effects of rooibos were clearly observed at 2 h of semen incubation reaching the best effects at 48 h, for example, with an increase of 30% in VSL (FR2 treatment). Regarding to this parameter, Holt *et al*. [29] reported that spermatozoa from the most fertile boar ejaculates tend to swim with straighter tracks (i.e., spermatozoa with higher LIN and VSL). In the same study, the authors also found positive relationships between litter size and VAP, STR, and VSL, which in our study were significantly increased by several rooibos treatments. Rooibos extracts also improved SMI and PM up to 96 h of semen storage. The importance of this result could be reflected in the fact that sperm with a defective or low progressive motility are mainly found in the female backflow after AIs [30, 31]. In other studies using rooibos aqueous extract as a drinking water in rats, Opuwari *et al*. [13] did not find significant differences in sperm kinetic parameters, although sperm concentration, motility, and viability showed higher values than those of the control group [12, 13]. In this way, our results provide further support to the positive effects of rooibos extracts on several parameters related to sperm function.

Overall, we found protective effects of rooibos treatments on the acrosome structure from 48 h of semen storage. Several studies have shown the importance of the acrosome integrity in fertility trials [32–34] highlighting the importance of our results. Similar results were found using rosemary (*Rosmarinus officinalis*) aqueous extract on post-thawed boar sperm [20]. Related to the sperm membrane integrity, even though for most rooibos treatments the percentages were higher than the control group, we only found significant differences in one treatment (UR4) at 48h. Further analyses need to be performed in order to confirm our results, such as measuring the levels of sperm LPO under an induced oxidative stress. In this regard, Awoniyi *et al*. [11] found that fermented rooibos aqueous extract, administered as drink water, significantly decrease the LPO levels in the testes of oxidative stress-induced rats. In a similar study in rat epididymal sperm, the catalase activity was enhanced under consumption of rooibos aqueous extract [12].

In the present study, we found higher TPC and TAC in the UR than in the FR aqueous extracts, as previously reported in several studies [3, 4]. Our results related to the TPC of rooibos are within the ranges previously reported [3, 12]. The fermentation of rooibos leaves and stems induces quantitative changes in the phenolic composition (reviewed in: [1]). Even though the total flavonoid content in aqueous extracts is higher in the UR, some of them (like flavonols) are higher in the FR [12, 13]. As inhibitor of tumor promotion in mouse skin, soluble fractions from the FR extracts had stronger effects than the UR, despite the fact that the latter has higher TPC than the FR [35]. In the present study, we found better results in terms of sperm velocity in the FR treatments, that is, higher number of these treatments had positive effects on sperm kinetics in comparison with the UR treatments. Moreover, VAP was significantly increased only in the FR treatments. This fact could be explained by the higher content of quercetin found in the FR in comparison with the UR aqueous extracts [3]. It has been reported that within the flavonoids found in the rooibos aqueous extracts, the quercetin has the greatest capacity as a free radical scavenger [4]. Thus, the flavonol quercetin has shown protective effects on human, rabbit, and bull spermatozoa by scavenging ROS [36–38]. Furthermore, Awoniyi *et al*. [11] found an increase in SOD activity and a reduction of LPO in the testicular homogenate of oxidative stress-induced rats fed with the FR aqueous extract. On the contrary, the authors did not find any positive effect on the same parameters using the UR extract. All these studies may explain the best results obtained in our study by using the FR extracts on boar sperm: the higher quercetin content in the FR aqueous extract could better protect against LPO and also increase the activity of the SOD in boar sperm. Nevertheless, further analyses are needed in order to confirm our hypotheses.

Our results revealed an optimal concentration range of TPC from 4.5 to 11.5 μg/ml (from R4 to R2) for boar semen, which is similar to the one used to stimulate osteoblast activity [7]. Nevertheless, the concentration of 11.5 μg/ml (R2) was suitable till 48 h due to the pronounced decrease in sperm motility found at 96 h of semen storage. On the other hand, we found that the highest concentrations of rooibos extracts (TPC: 27 μg/ml) were not suitable for boar sperm given that from 2 h of sperm storage significantly decreased the percentage of motile sperm. This decrease of sperm motility was more accentuated during semen storage with less than 30% and 10% of motile sperm at 48 h for the FR and the UR, respectively. It is well known that low levels of ROS are necessary for a normal sperm function and that the balance between the amounts of ROS produced and scavenged promote or jeopardize a given sperm function [39–41]. It could be possible that in our study, the extract with the highest proportion of rooibos partially inhibited sperm motility due to an excessive ROS scavenging activity rather than a cytotoxic effect. Supporting this hypothesis, the FR1 and UR1 treatments did not show significant differences in the sperm membrane integrity compared to the control group. Furthermore, the percentage of undamaged acrosome as well as the value of some kinetic parameters (i.e., LIN, STR, and VSL) was significantly higher than those of the control group.

## Conclusions

In conclusion, we provided empirical evidence that the rooibos extract enhances sperm velocity, protects the acrosome structure, and tends to preserve the membrane integrity up to 96 h of boar semen liquid-storage. Moreover, we have fitted the optimal concentrations of the rooibos extracts for the boar semen, obtaining the best results using the fermented type. The influence of the different flavonoids present in the rooibos extracts on sperm characteristics is not known at present and needs to be evaluated. Our next step is toward investigating the effects of the rooibos extract on sperm function under induced oxidative stress as well as in sperm fertilization ability. The rooibos extract represents a cheap and natural source of antioxidants for the preservation of boar semen.

## Supporting information

S1 Dataset Total polyphenol content and total antioxidant capacity in the rooibos aqueous extracts. (XLSX)

S2 Dataset Boar sperm parameters during semen storage at 17°C. (XLSX)
